# Can Children Have Ordinary Expectable Caregiving Environments in Unconventional Contexts? Quality of Care Organization in Three Mexican Same-Sex Planned Families

**DOI:** 10.3389/fpsyg.2018.02349

**Published:** 2018-11-30

**Authors:** Fernando Salinas-Quiroz, Fabiola Rodríguez-Sánchez, Pedro A. Costa, Mariana Rosales, Paola Silva, Verónica Cambón

**Affiliations:** ^1^National Pedagogic University, Mexico City, Mexico; ^2^William James Center for Research, Instituto Universitário de Ciências Psicológicas, Sociais e da Vida, Lisbon, Portugal; ^3^Independent Consultant, Mexico City, Mexico; ^4^Institute of Psychology, Education and Human Development, University of the Republic, Montevideo, Uruguay

**Keywords:** modern families, gay fathers, lesbian mothers, parenting styles and practices, attachment, sensitivity, quality of care, collective case study

## Abstract

The aim of this research was to explore the elements that configure the quality of care among three Mexican same-sex planned families: two female-parented families (through donor insemination) and a male-parented one (through adoption). The first family consisted of two mothers and a 3-year-old daughter; the second one had two mothers and a 1.5-year-old set of boy twins and the third family consisted of two fathers and a 2-year-old girl. It was assumed that Ainsworth’s notions of quality of care organization are useful in order to understand caregiver–child attachment relationships, regardless of the parents’ sexual orientation. A collective case study was selected due to the fact that these families shared their “unconventionality” (i.e., parents were not heterosexual) and the fact that they were planned, but each one constituted a particular case with a unique configuration. Four trained independent observers used the q-sort methodology (Maternal Behavior Q-Sort and Attachment Q-Sort) to describe parents’ and children’s behavior, respectively. The findings showed that parents were highly sensitive and all children used them as a secure base. To provide an in-depth examination of which elements configure the quality of care, a semi-structured interview with each parent was carried out. Through a thematic analysis, an over-arching theme named *Affections and Emotions* was identified, together with six subthemes: *(1) Creating an affective environment; (2) Being available; (3) Acknowledging and expressing emotions; (4) Perceiving, interpreting and responding adequately to the child’s real self; (5) Taking the child’s perspective into account;* and *(6) Agreeing on roles and dividing the tasks*. In order to showcase the particular configuration of gay parenting, the male-headed family narrative is reported in detail, because gay parents have been perceived as violating traditional gender roles as well as the hegemonic model of masculinity. The findings were consistent with the notion of quality of care as proposed by Ainsworth and her collaborators. The implications of the methodological device and research regarding same-sex planned families are discussed so as to understand the organization of the caregiving environment.

## Introduction

In 2003, Mexico’s Federal Law to prevent and eliminate various kinds of discrimination was enacted, facilitating the approval of the Civil Union Act in Mexico City in 2006. These legislative acts paved the conditions for both the 2009’ legalization of same-sex marriage and the possibility of child adoption among same-sex couples, being enforced the following year. Mexico City was the second megalopolis in Latin America to do so after Buenos Aires, Argentina (Díez, 2013). In June of 2015, the Supreme Court of Justice of Mexico decided that denying same-sex couples the right to access civil marriage was unconstitutional, thus mandating each State to allow same-sex marriage. However, every single State has to produce legislation to that effect, and to this date only 12 out of 32 States in Mexico have done so. In 2016, the Mexican President proposed to introduce equal access to marriage into the Federal Civil Code, but, after months of social upheaval, this effort failed to be approved.

According to Medina (2015), by the end of July of 2015, only eleven same-sex couples were able to legally adopt children in Mexico: seven in Mexico City, two in Coahuila and two in Yucatán. Nevertheless, the exact number of Lesbian and Gay (LG) parents in Mexico remains uncertain due to the existence of other possible family configurations (e.g., donor insemination or previous heterosexual relationships). Based on 2014 data from the Ministry of Social Development (*Secretaría de Desarrollo Social*), the National Population Council (*Consejo Nacional de Población*), the National Institute of Statistics and Geography (*Instituto Nacional de Estadística y Geografía*), the Institute for Social Research at the National Autonomous University (*Instituto de Investigaciones Sociales de la Universidad Nacional Autónoma de México*) and The College of México (*El Colegio de México*), it was estimated that there were 250,000 same-sex “nuclear families” in Mexico, within which 172,000 (68.8%) had children (Giraldo, 2015).

Careaga (2011) claimed that in Mexico the majority of LG individuals with children, became parents in the context of a previous heterosexual relationship which is consistent with the existing international research (e.g., Patterson and Tornello, 2010; Tornello and Patterson, 2015). However, “a generational change in timing and pathways to parenthood is taking place” (Tornello and Patterson, 2015, p. 44), because younger LG individuals are having children after coming out, through sperm donation (Lingiardi et al., 2016), surrogacy (Blake et al., 2017; Carone et al., 2017, 2018), adoption (Goldberg, 2012), informal adoptions (Amazonas et al., 2013), step parenting and co-parenting (Tornello and Patterson, 2015; Carneiro et al., 2017).

Patterson (2009) stated that LG individuals and same-sex couples were capable of meeting child’s best interests; therefore, they should be granted the same rights and accept the same responsibilities as heterosexual parents. In 2005, The Committee on the Rights of the Child stated, in the General Comment No. 7, that for the exercise of their rights, children have particular requirements for physical nurturance, emotional care and sensitive guidance. Through attachment to their parents “[…] children construct a personal identity and acquire culturally valued skills, knowledge and behaviors. In these ways, parents (and other caregivers) are normally the major conduct through which young children are able to realize their rights” (United Nations Committee on the Rights of the Child [UNCRC], 2005, p. 8).

Since 2010, same-sex couples are able to legally adopt children in Mexico City. New planned family configurations open up new possibilities to carry out naturalistic observations of cild–caregiver exchanges since early childhood (Carneiro et al., 2017). John Bowlby revolutionized our thinking about a child’s tie to the caregiver and its disruption through separation, deprivation, and bereavement, Mary Ainsworth tested some of Bowlby’s ideas empirically and contributed with the concept of the attachment figure as a secure base from which an infant can explore the world (Bretherton, 1992). Child–caregiver relationships during the first years of a child’s life are considered central for their healthy development, and “the relation between quality of care […] and child attachment security is a cornerstone of the Bowlby-Ainsworth perspective” (Posada et al., 2016, p.297). While Bowlby outlined the broader principles of attachment theory, and highlighted the child’s side of the relationship, Ainsworth concentrated on dyadic exchanges. Her observations led her to conclude that the organization of parental behavior is central to a child’s sense of security and his or her ability to use their caregivers as a secure base (Posada, 2013).

Throughout the meticulous examination of her own narrative records, describing different child–caregiver interactive behaviors, Mary Ainsworth identified the sensitivity construct (Bretherton, 2013). Sensitive parenting is defined as the caregiver’s ability to perceive child signals, to interpret them correctly, and to respond to them contingently and appropriately (Ainsworth et al., 1974). Sensitivity implies decoding and helping children express their feelings and experiences using words. Beyond responding to a child’s need for care and affection, sensitivity also involves respect for the infant as a valuable person who has autonomous needs, feelings and a mind of his or her own. Accordingly, what the child communicates is seen as worthy of attention, understanding and adequate response (Grossman et al., 2013) which relates entirely to a human rights perspective.

Ainsworth’s insights about caregiving, quality of care, and methodology are a baseline for research into the caregivers’ contribution to their relationships with children (Posada, 2013), regardless of their sexual orientation. She identified some centerpieces of the “ordinary expectable caregiving environment” (Bowlby, 1969/1982) upon which affective bonds rely upon. Children whose parents are commonly accessible at a physical and a psychological level, sensitive to their communications, accepting of their needs, and cooperative, are able to anticipate that their caregivers are accordingly responsive to their initiatives. The main feature of this environment is the quality of mutual delight in the dyad, including the caregiver’s ability to express it verbally (Ainsworth, 1967; Bretherton, 2013).

Mary Ainsworth concluded that over time, what matters the most, is the organization and patterning of parental behavior when looking for qualitative differences in child–caregiver attachment relationships (Posada, 2013). “[…] the dyadic patterning of […] ‘behavioral conversations’ form […] the basis for infant’s developing personality structure” (Bretherton, 2013, p. 464). Ainsworth (as cited in Bretherton, 2013) followed a “back-and-forthing” procedure where she examined her previously collected narrative records in relation to each Strange Situation Procedure group. This process helped her develop a set of four scales that reflect key qualities of behavior in caregiving routines: (1) Sensitivity–insensitivity to the baby’s signals and communications; (2) Cooperation–interference with the baby’s ongoing behavior; (3) Acceptance–rejection of the baby’s needs, and (4) Accessibility–ignoring. These scales were used to conceptualize maternal care (Ainsworth et al., 1971). Ainsworth et al. (1978) discovered that the first scale (sensitivity–insensitivity) was highly correlated with attachment security scores. Moreover, sensitivity has been identified as an important parental characteristic which facilitates secure attachment in children (De Wolff and van Ijzendoorn, 1997; Atkinson et al., 2000; Bakermans-Kranenburg et al., 2003; Moran et al., 2011). This finding is precisely one of the main reasons why researchers subsequently focus on, and use, the sensitivity construct when referring to the *quality of care* (Posada and Waters, 2014).

The relational focus of this construct was underlined by Ainsworth (1967) when she stated that sensitivity to signals involve playful interaction with children, suggesting that adults’ caregiving must be attuned to the child’s own timing. The sensitivity–insensitivity to the baby’s signals and communications scale highlights the emotions of both members of the dyad. Parents with high ratings on this scale are expected to have insight into their own mood and its effect on their child. Even though sensitivity is not synonymous with positive affect, and can be accompanied by low responsiveness (Mesman and Emmen, 2013), the combination of positive affect and high responsiveness is characteristic of the sensitive caregiver (Mesman et al., 2015). Beyond intellectual awareness of cues, Ainsworth (1970) proposed that a caregiver ought to feel things from the child’s point of view before he or she is able to respond accordingly. Consecutively, the adult learns about the appropriateness of his or her responses from their child’s feedback, which leads the caregiver to cease or persist.

“Far from a set of fixed attributes that (caregivers) may display on all occasions, specific (parental) sensitive behavior seems tailored to the particular situation and condition of the child” (Posada, 2013, p. 646). Ainsworth and Bell (as cited in Bretherton, 2013) declared that both, caregivers’ and child’s contributions to the interaction have spiral effects where it is impossible to identify independent from dependent variables as the emphasis is placed on the organization of specific forms of caregiver’s behavior in coordination with that of the child, without pursuing a causal model. Bowlby’s and Ainsworth’s emphasis on caregiver-child experiences and how they mutually influence each other when exchanging, the organization and patterning of parental behavior, and the secure base concept as an organizational construct, are extremely valuable lessons (Posada and Waters, 2014).

Summarizing, Ainsworth’s notions regarding *quality of care* organization are a cornerstone for research on child–caregiver attachment relationships irrespective of parents’ sexual orientation. Research on same-sex planned families enable developmental psychologists to explore particular adaptations of the caregiving behavioral system to different types of family configurations; however, previous studies on same-sex parenting have focused on children’s developmental outcomes, leaving aside naturalistic observations of child–caregiver exchanges since early childhood.

In a meta-analysis of developmental outcomes for children of 564 same-sex and 641 heterosexual parents, the mean age of children represented in the studies was 10.4 years with a range of 5–24 years (Crowl et al., 2008). In a more recent meta-analysis, participant’s ages ranged from 2.92 to 24.91 for the same-sex group, and from 3 to 24.46 for the different-sex group (Fedewa et al., 2015) therefore, little attention has been paid to very young children (0- to 3-year-olds).

Of the studies located in the latter meta-analysis, Averett et al. (2009) research did assess 1.5- to 5-year-old adopted children, but the researchers explored the extent of emotional and behavioral problems, and not the quality of the parent–child relationship. On this topic, Brewaeys et al. (1997) and Golombok et al. (2003) assessed it interviewing parents of 4- to 8-year-old children (of heterosexual conception and donor insemination). A decade later, Golombok et al. (2014) used The Etch-A-Sketch task (Stevenson-Hinde and Shouldice, 1995) and The Coconstruction task (Steele et al., 2007) as observational instruments based on attachment theory, to assess parent-adopted child’ interactions (3- to 9-years-old), through structured activities over short periods of time (drawing and building blocks). Perry et al. (2004) administered a structured doll play technique (The MacArthur Story Stem Battery) in order to examine the internal representations of children with different attachment relationships. Participants were 5- to 9-year-old children (of heterosexual conception and donor insemination) raised in lesbian-mother planned families compared with children in two-parent and single-parent heterosexual families. In all cases, positive mother–child relationships and well-adjusted children were found.

Comparative studies were necessary to demonstrate that the psychosocial development of children raised by same-sex parents were not negatively affected by their parents’ sexual orientation, and that LG people were as capable at parenting and child caring as heterosexual couples (Crowl et al., 2008; Biblarz and Savci, 2010; Fedewa et al., 2015; Carneiro et al., 2017). Nevertheless, comparing heterosexual-parented families with same-sex planned families reinforce heteronormativity by using heterosexual-parented families as the ideal, undermining the particularities of same-sex planned families. Clarke (2002) recommended that researchers should study the unique processes and challenges of same-sex planned families, particularly through qualitative and mixed methodologies. Observational assessments based on attachment theory have been used to study the quality of same-sex parent–child relationships, nevertheless, studies have focused exclusively on preschoolers and older children (e.g., Golombok et al., 2003, 2014). Therefore, the aim of this research was to explore the elements that configure the quality of care among three Mexican same-sex planned families with 0- to 3-year-old children, integrating quantitative and qualitative data.

## Materials and Methods

This research utilized a collective case study (Stake, 2008) as same-sex families share their “unconventionality” (i.e., that they are not heterosexual), but each one constituted a particular case with a unique configuration. Parents’ caregiving behavior (also known as sensitivity) and children’s organization of secure base behavior (also known as security) have been assessed using the Q-sort methodology through naturalistic observation of infant-parent interactions, but only with heterosexual parents. The Attachment Q-Sort (AQS) and the Maternal Behavior Q-Sort (MBQS) were chosen as standardized quantitative measures, as well as a semi-structured qualitative interview, to explore the elements that configure the quality of care.

### Participants

Parents were contacted through a Lesbian, Gay, Bisexual and Trans (LGBT) non-governmental organization (NGO)^1^. The only inclusion criteria for study entry were same-sex couples living together with offspring younger than 6 years of age. The NGO representative extended the invitation to the 703 Facebook group members^2^. Eight same-sex planned families agreed to participate. They were divided into two groups according to the developmental stage of their children: 0- to 3- and 3- to 5-year-olds. Each group had four families. For the present study, only families from the first group were considered. It was not possible to interview one of them; consequently, only three same-sex planned families from an upper-middle socioeconomic status participated under informed consent. Children mean age was 27.3 months old (*SD* = 8.5); parents mean age was 36 years old (*SD* = 6.6), with at least a college degree. The first family consisted of two mothers and a 3-year-old daughter (through donor insemination). The second family had two mothers and a 1.5-year-old set of boy twins (through donor insemination). The third family consisted of two fathers and a 2-year-old adopted girl (see Table 1). The sampling procedure was non-probabilistic and purposive (Flick, 2004).

**Table 1:** Demographic characteristics.

	Parents				Child
Mother/Father	Age	Education	Occupation	Relationship length	
Lilia	47	Bachelor	Yoga teacher and radio newscaster	6 years	Julia
Flor^∗^	41	Postgraduate	Yoga teacher and documentary film editor		36 months
					Gabriel and Paulo
Gabriela^∗^	37	Postgraduate	Self-employed	10 years	
Eugenia	30	Bachelor	Sales		19 months
Noe^∗∗^	30	Bachelor	Medical representative	2 years	Karina
Gerardo^∗∗^	35	Postgraduate	Physician		27 months

*^∗^Biological mother through donor insemination; ^∗∗^ Adoptive parent*.

### Procedure

After initial contact with the couples, each parent was individually observed and videotaped interacting at home with his or her child over a period of 2 h, as previous Ibero-American studies have endorsed (e.g., Monteiro et al., 2010; Posada, 2013; Posada et al., 2016, 2013, 2002). Mothers and fathers were told to go about their activities as they normally would. Observers were allowed to interact naturally with the mother/father and children at home. Parents’ and children’s behavior were reported immediately after the visit by four independent and Q-sort trained observers (two for the MBQS and two for the AQS). Afterward, in a second meeting, a semi-structured interview was conducted individually, i.e., without the presence of either the partner or the child. This procedure was repeated for each parent.

### Ethics Statement

Ethical approval for this study was obtained by the first and second author’s university Ethics Committee. Parents were informed that their participation in this study was confidential and provided both verbal and written consent before their participation. Parents’ names presented in this study are pseudonymous, and their identities are anonymous. All performed procedures were in accordance with the ethical standards of the institutional and/or national research committee and with the 1964 Helsinki declaration and its later amendments or comparable ethical standards.

### Measures

Since the AQS and the MBQS share the same methodology, it is crucial to understand this research method. The Q-sort methodology features a number of structural and procedural elements that distinguish it from more traditional approaches and make it more apt to produce a comprehensive and meaningful description of the observed interaction (Moran et al., 2011). Each individual Q-sort card contains a detailed single item or descriptor of a distinctive aspect of the parent–child interaction quality, and each Q-sort (either the AQS or the MBQS) comprises 90 items. The objective of the sorting exercise is to describe the caregiver-infant interactions by corresponding each of these descriptors to scores ranging from 1 (“extremely uncharacteristic”; e.g., MBQS Item 2 “Unaware of, or insensitive to baby’s signs of distress”) to 9 (“extremely characteristic”; e.g., AQS Item 21 “Child keeps track of mother’s location when he plays around the house. Call her now and then; notices her go from room to room. Notices if she changes activities”) through a forced distribution.

The observers who collected the data were trained in the use of both Q-sorts (AQS and MBQS separately). Training for each Q-sort consisted of first reading and discussing the meaning of the 90 items. This was followed by three to five practice observations and q-descriptions of parental (MBQS) and child behavior (AQS) during videotaped child–mother interactions at home/park. Trainee observers’ descriptions were compared to those of an expert; an observer was considered trained when she or he obtained an inter-observer reliability with an expert (i.e., correlation corrected for number of observers using the Spearman–Brown formula) of at least 0.80 in three practice observations.

#### Parental Behavior (Sensitivity)

The MBQS (Pederson and Moran, 1995) comprises 90 items, which were based on Ainsworth’s conceptualization of quality of care. Data in support of its validity has been reported elsewhere (e.g., Pederson et al., 1990; Moran et al., 1992; Pederson and Moran, 1995, 1996). The MBQS was originally designed for home observations of maternal interactions with infants, but it has also been used with fathers (e.g., Colonnesi et al., 2013). Its construct validity has been demonstrated by meta-analytic data showing its predictive capacity with respect to child attachment security (Atkinson et al., 2000; van IJzendoorn et al., 2004). Mean inter-observer reliability index (calculated from the agreement between q-descriptors from independent observers; Block, 1978) was 0.93 (range = 0.87–0.97).

#### Secure Base Behavior (Security)

Children behavior during interactions with their parents was described using the AQS (Waters, 1995). It also comprises 90 items that assess the organization of children’s attachment behavior in naturalistic settings. The 3.0 version of the AQS represent a subset and an extension of the 100-item pool described by Waters and Deane (1985). “While multiple observers, multiple settings, and multiple occasions constitute the ideal, in practice researchers have reported reliable and valid assessment under less than ideal circumstances (e.g., single observers or single occasions)” (Waters, 1995, p. 234). Its validity has been documented elsewhere (e.g., Pederson and Moran, 1996; van IJzendoorn et al., 2004; Posada et al., 2013; Cadman et al., 2017). In a recent meta-analysis, the mean security score was 0.35 (Cadman et al., 2017) which is comparable to the mean security score of 0.31 reported in the 2004 meta-analysis (van IJzendoorn et al., 2004). Mean inter-observer reliability index (calculated from the agreement between q-descriptors from independent observers) was 0.73 (range = 0.41–0.94).

#### Semi-Structured Interview

To provide an in-depth examination of the elements that configure the quality of care, semi-structured interviews (Flick, 2004) were conducted and audio-recorded. The interviews lasted 2 h on average and consisted of 38 questions focusing on parenting and childrearing practices. They included eliciting questions such as “what is a normal week day like?,” but also questions that addressed the following topics: parents’ couple relationship quality; shared activities between family members; rule-setting; sleep, feeding, bathing and bladder control; play; temperament and personality, among others.

### Analysis and Interpretation of Data

The most common measure associated with the q-sort methodology is the Pearson’s *r* value describing the item-by-item correlation between the sort describing a particular dyad interaction and the aggregate sort of the prototypical secure children (AQS) and/or prototypical sensitive caregiver (MBQS) performed by a group of experts in attachment theory. The MBQS and the AQS were used to describe parents’ and children’s behavior, respectively.

Qualitative data (i.e., transcripts of interviews) regarding quality of care were analyzed using Thematic Analysis (Braun and Clarke, 2006), through an inductive or ‘bottom up’ approach, which meant that themes were identified based on the raw data, without a theoretical frame or prior coding system, and followed a constructionist paradigm. Despite the fact that a ‘top down’ approach informed by previous theory could have been utilized, it was decided to use an inductive approach “…because it allows a freer and richer description of the data as well as the possibility of capturing more nuanced relationships between different meanings that a more rigid approach could miss” (Costa and Tasker, 2018, p. 11). The first, second, and fourth authors read, analyzed and performed the initial editing and coding of all transcripts, and these codes and the main themes identified within the data were discussed with the third author. Once this task was completed, the first, second, and fourth authors again actively engaged in a discussion about the themes in order to clarify the specificities and relations between them. Through a constant iterative process, the third author reviewed the initial codes and themes. This was followed by a discussion between the four of them about the naming and defining of the final themes presented in this analysis. Summarizing, investigator triangulation was achieved in all the analysis procedures to develop a broader and deeper understanding (Flick, 2007).

It was assumed that the analysis of similarities and differences between each family would enable a better understanding of the quality of care organization in same-sex planned families. The first part of the results focuses on the six subthemes that were identified for the three participant planned families. Afterward, the narrative (Zeller, 1998) of the male-headed family is displayed due to the richness of the data that this couple provided and to showcase the particular configuration of gay parenting. Gay men as parents seem to challenge gendered and parenthood expectations and are judged more harshly than lesbian mothers because they are perceived as violating traditional gender roles and the hegemonic model of masculinity (Connell, 2005; Stacey, 2006; Wells, 2011).

## Results

### Quantitative Data

The mean score for the MBQS was 0.74 (see Table 2), which is comparable to the mean reported for Colombian (*M* = 0.69; Posada et al., 2004) and Uruguayan heterosexual mothers (*M* = 0.78; Silva, 2015). This score reflects that the same-sex parents that participated in this study perceived child signals, interpreted them correctly and responded to them contingently and appropriately. Regarding security, the mean score was 0.58 (see Table 2), higher when compared to a recent study that assessed Colombian, Mexican immigrants to the United States, Peruvian and United States children from heterosexual families, where the mean security score was 0.39 (Posada et al., 2016). Furthermore, the mean score for security (*M* = 0.58) was also higher than Posada et al. (2013) cross-cultural study means (*M* = 0.32 for Colombia, *M* = 0.30 for Peru and *M* = 0.45 for Portugal). This score reflects a smoothly functioning child–parent bond, and that children remarkably used both of their parents as a safety heaven, as well as a base from which to explore their surroundings.

**Table 2:** Sensitivity and security.

Parent	Child	Sensitivity (MBQS)		Security (AQS)	
Overall sample					
*M (SD)*		0.74 (0.25)		0.58 (0.19)	
Range		0.67–0.83		0.35–0.73	

Lilia	Julia	0.83		0.73	
Flor		0.79		0.66	

Gabriela	Gabriel and	Gabriel	Paulo	Gabriel	Paulo
Eugenia	Paulo	0.73	0.71	0.55	0.64
		0.70	0.67	0.63	0.57

Noe	Karina	0.73		0.35	
Gerardo		0.77		0.49	

### Qualitative Data: Thematic Analysis

Transcripts were coded without trying to fit into a pre-existing coding frame or analytic preconceptions (i.e., data-driven). On average, 438 inclusive and comprehensive codes were generated for each transcript (570 for Noe; 501 for Gerardo; 326 for Lilia; 334 for Flor; 446 for Gabriela and 452 for Eugenia). Eighteen internally coherent, consistent and distinctive themes were identified. Themes were named and defined by the first, second, third and fourth authors; all relevant extract for each theme were collated and themes were checked against each other and with the original data set. One theme, labeled as *Affections and Emotions* contained the greatest amount of codes. Such theme encompassed codes where parents expressed discernment, ideas and beliefs regarding their bond with their children, as well as situations or activity descriptions where perceptions and interpretations of child related emotions were reconstructed. Furthermore, this theme encompassed six often closely related subthemes: *(1) Creating an affective environment; (2) Being available; (3) Acknowledging and expressing emotions; (4) Perceiving, interpreting and responding adequately to the child’s real self; (5) Taking the child’s perspective into account;* and *(6) Agreeing on roles and dividing the tasks*. These subthemes are defined on Table 3, along with the specific elements for each planned family.

**Table 3:** Quality of care subthemes.

Subtheme	Definition	Lilia and Flor	Gabriela and Eugenia	Noe and Gerardo
Creating an affective environment	Providing a sense of love and protection to the child through physical contact and verbal expression and/or during play, daily routines and learning situations	Storytelling to the girl about her story Communicating through gazing	Pampering the children with food and play Rejoicing in twin’s achievements	Constantly telling Karina that they love her Tickling her and rolling Fostering a loving and guarding circle

Being available	Organizing schedules in order to be present and accessible to their children	Involving and giving undivided attention during playtime	Organizing flexible schedules for the children’s benefits	Seeking for free time in order to be with her

Acknowledging and expressing emotions	Becoming aware of their own emotions and the importance of teaching their children how to identify them	Recognizing when one is tired Respecting Karina’s negative emotions	Avoiding yelling at the twins so they do not get scared Setting limits when the boys hit each other	Balancing annoyance and irritation toward tantrums vs. coherent consequences (“time out”)

Perceiving, interpreting and responding adequately to the child’s real self	Remaining attentive to signals and interpreting the child’s negative behaviors in a flexible way in order to respond satisfactorily	Conceptualizing crying as something necessary Self-restraining and approaching to the girl gradually	Differentiating perceptions and making adjustments with Paulo and Gabriel Setting rules and limits that are aligned with the children’s level of development	Testing hypotheses to get to the cause of the girl’s annoyance Diverting attention during tantrums

Taking the child’s perspective into account	Recognizing and respecting the differences between the children and recognizing their worth for their own rights	Explaining the rules Respecting her own individuality Allowing her to choose	Making attributions and explanations for the twins, “even when they do not speak yet” Respecting their children’s needs (e.g., nap time)	Fostering autonomy by assigning tasks and responsibilities that are aligned with her age Negotiating and providing explanations

Agreeing on roles and dividing the tasks	Consenting and organizing to take differentiated roles between the couple and for the upbringing duties along with a support network	Flor taking on most of the upbringing activities	Involving in different activities (driving them to school, shower time, putting them to bed) even though Eugenia works business hours	Gerardo playing the role of the family’s main provider

#### (1) Creating an Affective Environment

The first common element was the importance parents bestow upon making their children feel loved and protected, which was expressed through kissing, hugging, pampering, massaging and tickling, as well as by using tender words or explicit verbalizations: “Flor and I told her the story of how we wanted to have a baby, and how we always wanted her, and how when she was born we were so joyful, and we always are” (Lilia). The importance participants placed on enjoying the interactions and communicating what their son/daughter means to them was also noticed, even only with a gaze: “I kiss her a lot, I hug her whenever I find a chance…I feel she gets me more through eye-gazing so I try, when speaking to her, to look her in the eye, laugh with her” (Lilia).

It is through playtime that adults “spoke the language” of the child, and in turn they used their imagination, played roles, and were able to interact more rough-and-tumble with them. Although in this study there is no deeper analysis of the practices and significance of playing, attention is drawn to the fact that playing was not only used as a strategy to divert attention during tantrums, or as something common among children at this age, but above all it was considered as an opportunity to interact, live together and foster the bond.

The positive and affective climate was generated not only during play or learning situations, but also in routines such as the nap. For Gabriela, the nap was of upmost importance, as she said that, when skipped, the twins could get in a bad mood. She also tried to “always have milk that is nice and warm,” as well as to “cook for them what they enjoy.” These expressions indicate how she brought significance and care into daily activities that might otherwise seem routine. Gabriela also described how she “shares all kinds of activities with Paulo and Gabriel on a daily basis,” as she “loves to discover her sons’ behaviors.” These mothers clearly illustrated how the hard work that comes with keeping and having daily routines does not prevent them from enjoying the upbringing process. Summarizing, it seems that among participants the premise was as follows: ‘let’s take advantage of every opportunity to show our love and to turn routine into playtime.’

#### (2) Being Available

Having flexible jobs and schedules was a characteristic of almost all participants whose aim was to be present: “be there” for their children both during play and when enforcing rules. Thanks to their support networks, they managed to share and divide tasks, giving priority to the attention they put on their children. Being available involved more than just a matter of organization; it had to do with undivided attention or the quality time allocated to the children:

“If I am playing with her I don’t allow anything to distract me -not using the phone, not doing anything else- as an adult it is very difficult sometimes [but] I feel that being involved with her during playtime is something she values a lot” (Lilia).

Creating an affective environment as well as the availability of the caregivers were neither an instinctive behavior nor a consequence of biological motherhood or fatherhood: participants’ stories revealed that it was a choice and a personal decision, and that once it was made it was taken with responsibility and dedication. Even in cases such as Noe or Lilia, who decided to take on the parental role when their partners had already decided to take it by themselves, it became evident how they “fell in love” with the girls (couples were not living together when both Gerardo decided to adopt Karina, and Flor decided to get pregnant with Julia). Accordingly, attachment bonds were developed and organized over concrete and ordinary situations, which can be described as patterns. The quality of the caregiving relies upon the continuity of the interactions.

#### (3) Acknowledging and Expressing Emotions

Parents were extremely thoughtful and conscious of their own strengths and difficulties about childrearing. They remained alert and vigilant toward their own mood, their couple functioning and any other aspect that they felt could interfere with their family life, as they were aware of how everything may affect the bonding. For instance, when Gabriela got upset and raised her voice, she immediately became conscious of how this impacted the twins (because they made “surprise faces”). So instead of preventing it just for the sake of what should or ought to be, she tried to avoid doing so because she could see Paulo and Gabriel’s reactions: “I don’t like to scare them…I’ve tried to avoid yelling.”

In that sense, they were adults who “got in touch with their emotions”; therefore, they taught their children the importance of doing so. Maybe this is the reason why, in general, they were so physical in showing love and worked hard toward creating caring environments. Thus, they were able to accept to themselves and admit to others (i.e., the interviewer) the emotions related to family upbringing such as annoyance, anger, fatigue, blame, and frustration, which are often culturally and socially denied (especially among mothers).

Parents became emotionally tense when enforcing rules and limits. This was reflected in many situations such as dealing with the annoyance that the daughter provoked when throwing the dish on the floor, while at the same time trying to remain firm about the “time out”; not telling the little girl off about not wanting to go to the bathroom, in order not to make her feel bad, while dealing with the frustration this situation generated; wanting to explain the world to the child, but also feeling so tired or stressed that you want to skip the explanation; finding the balance between wanting to prolong the playtime to see the daughter enjoying herself, but remaining strict about the sleep routine so that she gets enough rest; maintaining the balance between “showing too much love and care, and overcoming challenges or setting boundaries.” These statements were taken from the analysis of individual families and are not fully described in this paper. However, it was decided to include some of them, word for word, so as to exemplify the issue being discussed. The first two statements were made by Gerardo and Noe, while the others were made by Flor and Lilia. Regarding the statement “having to explain” to the child, Lilia admitted that she sometimes felt she did not want to explain anything and just wanted to say “because”! The last statements were made by Eugenia and Gabriela.

Loving the children and being available for them was not a synonym of denying oneself as a person; on the contrary, it allowed parents to acknowledge their own limits and to show their own emotions in order to teach empathy. As Lilia said: “Julia gives me a kiss when I’m upset.” These examples may well explain why, overall, affective behaviors as an over-arching theme was closely related to *Rules and Norms* (another over-arching theme). As Eugenia said: “when Paulo hits, we say *no, you don’t do that*, or we take him out of the crib,” as both mothers felt annoyed about brothers hitting each other. Therefore, love and care protected them from their own aggression and helped them develop self-regulation.

#### (4) Perceiving, Interpreting, and Responding Adequately to the Child’s Real Self

Overall, it was important to remain attentive as well as to interpret and respond to the child’s emotions, especially to the negative ones, as they tended to throw the adults off balance. Furthermore, allowing children to express themselves (through crying or tantrums) reflected the parents’ ability to integrate positive and hostile aspects without idealizing their children. Despite this, no parent attributed such behaviors to a personal matter, such as the child’s character or “just because.” This mainly was ascribed to developmental aspects and to the needs that required immediate attention (being hungry, tired or sleepy).

In regard to how to respond to specific behaviors, the overall agreement seemed to be: ‘depends on what triggered them.’ If it concerned overstepping a limit, then the parents were firm about imposing a consequence. On the other hand, if there were causes that justified the child’s being upset, then the parents diverted the infant’s attention, or simply gave the child some space and respected that feeling. As Lilia stated: “we must understand that Julia feels things… and that it’s not personal,” that is why sometimes they let her cry “as long as she needs to, [trying] to cut her some slack.” The following quotation is a clear example of sensitive parenting:

“I try to feel where her crying is coming from, and I sometimes feel that she needs to cry but she needs me to be there too, so I let her cry and try to approach her after some time until I feel she needs comfort and a hug; I try not to be hasty about her calming down, but to try and try physical contact until she welcomes the hug” (Lilia).

The repertoire of sensitive responses from the adults related to the development of the concept of knowing their child or children which was differentiated in the family with twins. These mothers’ reports seemed consistent, as they stated that Gabriel was a dominant and gifted child, and that at the beginning they felt that Paulo was developing more slowly, thus it makes sense that during the observation, Eugenia and Gabriela were more attentive toward the latter. This refers to the overall score in the MBQS which for both mothers was higher for Paulo than it was for Gabriel (see Table 2).

#### (5) Taking the Child’s Perspective Into Account

For the parents, remaining well informed helped them to identify what to expect during each developmental stage and realize that all children are different and develop at their own time. For example, Eugenia believed that “it is easier to calm them down now than when they were little, because now they understand better.” The shared concern with Gabriela over “Paulo’s lack of attention” reflected the understanding of how each child developed at a different pace, and elicited a response from the son, as she considered that both sons were attentive and such responses were differentiated. In this regard, it is noteworthy the wide knowledge each participant had about their children. While the adult who spent more time with the infant was the one who gave out more details about the child’s personality, it is evident that good communication existed in order to keep the other parent informed about the child’s doings.

Overall, there was a shared value of respect for the individuality of each children, whom were asked for their opinion on specific situations where they could undertake small decisions, such as combing their hair, getting dressed, deciding what to eat, what book to read, and so on. This shows how the focus was centered on the children, who were perceived as worthy of their own rights. These parents constantly sought the balance between making the children feel good, without being overindulgent by giving in to every whim. They worked day by day on providing space and playful activities without failing to follow the rules and routines, which were established in agreement among the parents; they both respected them and intended for the children to anticipate consequences or understand the aim of certain practices (such as the “time out”).

To take time to explain things to the children indicated that, for the adults, they were worthy of such explanations. For instance, Eugenia explained how she “chatted” with Paulo and Gabriel, stimulating language production, despite the fact that they were too young to speak yet (19 months old), thus treating them as persons capable of having intentions by sharing her day-to-day life with them. It is noteworthy that the limited speaking skills of the twins did not hinder communication and comprehension, and that the interaction patterns were not symmetrical, as it was the adult’s responsibility to stimulate their children. These parents slowly involved their children in simple household chores and tasks, consequently fostering autonomy and self-reliance. There was a clear knowledge of the children’s rights, consistent with their childrearing practices.

#### (6) Agreeing on Roles and Dividing the Tasks

Behind the quality of care, organized and coordinated couples that agreed, listened to each other, and respected the reached agreements about their children’s upbringing were found. Mothers and fathers took on differentiated duties and prevented a role from falling more onto only one parent, even when sometimes one of the parents was the primary provider of the family, and sometimes the other. Therefore, each one was in charge of taking care of different duties and routines. Both parents acknowledged the importance of their partner’s work and created together a solid ground for their children not only when establishing limits and setting rules, but also in maintaining the family’s main premises: love and care, protection and respect.

For example, Eugenia and Gabriela were equally in charge of direct and indirect care, as well as of the teachings, discipline, play and outdoor leisure. It was Gabriela the one who usually fed and drove the children to school, and the one that dealt with their negative behaviors, as it was Eugenia the one who worked on a schedule, which prevented her from being at home more time: them both agreed on these facts. So, the task assignment depended more on the specific work-related situations (as it is within most couples nowadays) and not on the men or women’s roles established within society. Relying on support networks such as family, friends and domestic workers was essential for these couples in order to be able to focus their efforts on what was most important to them: their children.

### Male-Headed Family Narrative: Gerardo, Noe, and Karina

Gerardo had always wished to become a father, but it hadn’t been in Noe’s plans when they first started dating. It cannot be inferred whether the difference in their parenting aspiration echoed in distinctive behaviors toward Karina that reflected in her dissimilar security scores (i.e., 0.49 and 0.35 with Gerardo and Noe, respectively); however, it suggests that further examination of this relationship in future larger studies would be convenient.

A few months after having started dating, Gerardo and Noe had the opportunity to adopt Karina, and they did; since it was such an early stage in their relationship, it was particularly important that she felt “loved and cared for.” They saw themselves as very loving and eager to display physical contact; these findings were consistent with two characteristic items of their MBQS’s: 38 (*Displays affection by touching*) and 43 (*Kisses baby on head as major mode of expressing affection*). Further, they often hugged her, and called her sweet names such as “beautiful” and “princess.”

Love was also displayed in the form of fun activities, such as singing and dancing with her, tickling her and rolling: “I invent songs for her every night and she asks me to sing them” (Noe). Item 6 (*Interactions appropriately vigorous and exciting as judged from baby’s responses*), item 30 (*Plays games with baby such as peek-a-boo, patty cake*) and item 32 (*Provides age-appropriate toys*) of the MBQS of both parents were also placed on pile 9 “most like,” so this was also evident during the naturalistic observation. Noe stated that when Karina hugged him, he “felt good”; both liked to stay in bed with her after waking up, and made the most out of the daily routines.

The value placed on affection and support toward Karina was something they sought to foster among the people who surrounded her (school and family): “Karina’s circle must be very loving and guarding” (Gerardo); additionally, Noe was interested in Karina “bonding with her cousins and having confidants.” This loving environment was related to certain attributions about her: “Karina is a very happy girl; her personality is a reflection of how loved she feels. She knows that her parents are here to support her in any situation” (Noe). The “being there” was a statement that Gerardo agreed with, as he tried not to miss any moment with his daughter and to “be present” at every developmental milestone: learning to walk, her first words, bladder control, etc.

It was very important for both parents to figure out Karina’s signals. Gerardo looked for multiple possibilities to infer what was wrong with her when he perceived her different from her “normal”: it could be due to “lack of sleep, hunger or the need to go to the toilet,” so he related the girl’s unrest with physical discomforts. Once the previous hypotheses were disregarded, they showed great difficulty understanding her, or at least her negative behavior (crying, yelling, and tantrums). Gerardo said: “there are times that she simply doesn’t want anything and there’s no explanation.” Noe stated: “it’s frustrating not knowing what is wrong; not knowing what triggers it [the tantrum].” He felt quite helpless when he did not understand her. They both put continuous effort into trying to understand what went on with Karina. These data triangulated with the MBQS’s item 5 (*Notices when baby smiles and vocalizes*), item 9 (*Responds consistently to baby’s signals*), item 12 (*Interpret cues correctly as evidenced by baby’s response*) and item 60 (*When baby is distressed, is able to quickly and accurately identify the source*), which were all “extremely characteristic” in both parents during the observations.

Depending on the situation, they respected Karina if she didn’t want to do certain things, because “it is important not to exercise more control than necessary and give her space” (Gerardo). Other times when facing “communication blockages” (in their own words), they resorted to dialog. Noe and Gerardo told Karina: “look, we are not understanding each other, so calm down and we can then talk and see if we can understand each other better.” The uneasiness was not only brought by being unable to understand her, but they also got upset when Karina “*was not willing to*” do certain things; Gerardo “gets upset when Karina has an urge to pee but *refuses* to go to the bathroom and wets herself”; or when she “*refuses* to dress even when she’s been given 5–10 min” (italics added by the authors).

Taking into consideration that Karina was barely over 2 years old, it is likely that both parents overestimated the girl’s skills. Even though they remained up-to-date regarding child’s development and parenting aspects, there seemed to be a mismatch regarding the interpretations and expectations of Karina’s negative behaviors. Maybe the girl was showing specific needs through crying but the parents gave the impression of asking her to self-regulate at a very young age. All in all, Noe considered that “as any other child, she cries when she feels her parents’ absence,” and consciously understood that crying was a way of communicating and that separation causes anxiety.

The tantrum controversy was an iterative issue with both parents, who thought that Karina did “more tantrums than normal,” so they did everything in their power to figure out the causes. Noe thought that she wanted to call their attention, whereas Gerardo considered that it could be due to a physical problem. However, they both referred to her age as being the main reason for “so many tantrums,” so it would seem that, in the end, it was “normal.” Gerardo said: “she draws attention by crying, hitting or just by saying *no* [because] it is the only way for her to communicate right now; as any 2-year-old, it’s normal that she is impatient and demanding…it’s a difficult age.” Noe recognized that “she displays the characteristics of the ‘terrible twos.”’ Interpretations of specific situations such as these were related to wider judgments: Gerardo recognized that a child’s upbringing was an extremely demanding task and he assumed this without denying that “a child makes parents feel energy-depleted and also takes the energy out from the couple.”

For them, tantrums did not represent the only inadequate behavior to be addressed, as “there are rules that are non-negotiable: do not hit, throw food or cutlery on the floor, or display violence; do brush your teeth and get dressed” (Gerardo). Noe felt irritated by the same behaviors: “throwing the dishes or food onto the floor, and slapping.” They both agreed on the way the tantrums were handled, via a consistent use of “punishment” (Noe) or “time out” (Gerardo). It did not matter who carried out the disciplinary measure: if one did, the other one followed. Another strategy they resorted to was to divert her attention: “when Karina didn’t want to eat, Gerardo drew attention to the bubbles and the tantrum ended” (Noe).

Negotiating with Karina was important for both of them, not only as a strategy against the tantrum so that “she feels that she is winning or agreeing” (Gerardo), but also as a way of showing that they took her point of view into consideration. It was very important for the parents to understand Karina: asking her what was wrong or what was bothering her. When faced with a “communication blockage” or when asking her what she wanted or needed, Noe could not identify if “she can’t make herself understood or they don’t understand her.” The fathers admitted that Karina had needs; that communication was a two-way process, and they did not blame her for the lack of understanding. Creating a positive environment for Karina involved recognizing negative emotions in both, the parent and the daughter, in order to treat those with respect. Gerardo was interested in making Karina understand that there were times when he also needed “space” because he was upset, and he expressed it in a very explicit way due to the fact that he considered that the girl needed to learn that “people need a space when they are upset.” This request from father to daughter was associated with what Gerardo gave in return. While Gerardo “feels hurt when Karina gets angry or ignores him” he knows that he is “the adult, and would be foolish not to lower the bar when Karina is angry.”

Communication and understanding was key to these parents, not only interpreting what Karina wanted, but also talking and explaining things to her. Gerardo highlighted the importance of using a clear and understandable language, suitable for the girl’s age, when handling issues; for example, he explained to her why they made use of the “time-out” strategy, or that when leaving her at school, they needed to go and then come back to pick her up, or simply engaging in conversation while in the car. Consistency with another MBQS item was identified (item 79), which was characteristic in both parents: *Frequently repeats words carefully and slowly to the baby as if teaching meaning or labeling an activity or object*. All of these announcements and narratives acted as important elements for the child to anticipate events (Bruner, 1980): as Gerardo pointed out “eventually… she’ll have to know” the story and “[understand] that giving her for adoption was her mother’s act of love, as she wasn’t able to provide and take care of her” (Noe).

The need to explain things to Karina was linked to the parents’ concern regarding the future, as “she is a baby girl who can be exposed to discrimination due to her characteristics” (Gerardo), and that might be the reason why he did not want to over protect her, and why he “assigned” her easy house chores in order to encourage independence and autonomy. He allowed her to try to solve certain things by herself even when he knew she wouldn’t be able to, because “Karina is learning to face frustration”; hence, fulfilling all of her wishes and preventing her from getting upset was not among his educational goals. Such autonomy bore fruit and was acknowledged, for example, when Noe described the child as an “independent girl who tries to do her best even though things are not that easy, and she does not act like a spoiled child.” Noe said that both of them “try to involve her in everything: let’s cook, help us, and pass me this or that,” thus the every-day life became playful, shared and full of learning experiences.

It stands out how Noe detailed Karina more extensively, during the interview: “she is daring and fearless, but she exercises caution; she knows that when crossing certain streets, she must hold our hand; she is very feminine and active; she’s intelligent and very expressive; when we have guests over, she is somewhat shy, but then she gains trust and likes to draw attention.”

The fact that both parents were consistent does not mean that they knew or interacted with Karina in the same way; this can also be observed in any family dynamic, where parental roles are usually differentiated. As Gerardo pointed out, Noe played more the role of the “stay-at-home” parent, whereas he played the role of the “provider.”

Even though what being a gay parent means for them was not addressed in this analysis, it can be said that their difficulties and concerns showed the pressure they might feel to be perfect and to prove that they were good parents, mainly because they both were males.

## Discussion

The aim of this research was to explore the elements that configure the quality of care among three Mexican same-sex planned families with 0- to 3-year-old children. A collective case study design that comprised two different techniques was chosen. The MBQS and the AQS quantitative scores served as a benchmark for parents’ and children’s behavior, respectively, whilst the qualitative thematic analysis allowed a deepening of meanings and beliefs underlying the quality of care organization in these families. As stated earlier, comparing same-sex planned families with heterosexual-parented families reinforce heteronormativity by using heterosexual families as the ideal. Hence, Ibero-American studies that used the MBQS/AQS were only taken as a point of reference. Each of the three same-sex planned families constituted a particular case with unique processes and challenges (Clarke, 2002) but, at the same time, shared their “unconventionality”; that is, that parents were not heterosexual.

The mean score for the three same-sex parents’ sensitivity was 0.74. Therefore, it can be said that the LG caregivers that participated in this study perceived child signals, interpreted them correctly, and responded to them contingently and appropriately, in other words, the three same-sex caregivers displayed sensitive parenting. Security scores (*M* = 0.58) reflect that children outstandingly used both of their parents as a base from which to explore their surroundings, as well as a haven of safety-seeking proximity with them at different times and across contexts-, in other words, a smoothly functioning child–parent bond. Secure base relationships are hypothesized to be observable in children who have been exposed to ordinary expectable caregiving environments (Bowlby, 1969/1982).

Due to the lack of attachment theory-based studies in Mexico, there is no such thing as normative data. The only available baseline was research carried out in countries with common history, androcentric culture and high levels of gender-role traditionalism and religiosity (Steffens et al., 2015; Costa and Salinas-Quiroz, 2018). Related Ibero-American studies that assessed heterosexual parented-families reported similar sensitivity scores but lower security means. The organization of parental behavior is central to a child’s ability to use the caregivers as a secure base (Posada, 2013) and there is some evidence that socio-demographic factors predict sensitive parenting (Mesman et al., 2012, 2015). The three same-sex families that participated in this study belonged to a high socioeconomic status where parents had flexible jobs and schedules and were on their late thirties (*M* = 36). On the other hand, Colombian, Uruguayan and Portuguese heterosexual-parented families came from middle-class sectors (Posada et al., 2004; Monteiro et al., 2010; Silva, 2015; Posada et al., 2016) where mothers were younger (e.g., *M* = 32, Posada et al., 2016; *M* = 31.3, Posada et al., 2004) and worked outside the home. Peruvian and Mexican immigrant dyads came from a low socioeconomic background (Posada et al., 2016). All of the above-mentioned socio-demographic characteristics might explain the elevated security scores, since evidence highlights that younger mothers and low-income parents have less favorable parenting behaviors (Mesman et al., 2012; Scholmer and Belsky, 2012).

The thematic analysis of the qualitative data proved useful in order to provide a more in-depth analysis of the three same-sex planned families’ quality of care. This approach offered a freer and richer description of the data which was later contrasted with the literature. Themes and patterns were identified via an inductive ‘bottom-up’ approach (Braun and Clarke, 2006), a process that resembles Ainsworth (1967) “back-and-forthing” procedure. The six subthemes reflected patterns in the three same-sex parented families.

The subthemes *Creating an affective environment* and *Being available* both encompassed key elements of the quality of care just as previously conceived by Ainsworth (1967) as the six parents displayed the ability to take the child into consideration and to actively participate when interacting, particularly through playful and attuned exchanges with a sense of delight. The *Acknowledging and expressing emotions* subtheme referred not only to decoding and helping children verbally express feelings and experiences, but also to the fact that the contributions from caregivers and children to the interaction are caught up in an interacting spiral, as Ainsworth and Bell highlighted (as cited in Bretherton, 2013).

The *Perceiving, interpreting and responding adequately to the child’s real self* subtheme, encompassed three main elements of sensitivity, and, although a bottom-up approach was used to analyze the data, it is noteworthy that this subtheme corresponded exactly to how Ainsworth characterized the sensitivity construct. The *child’s real self* was highlighted within this subtheme because these parents were able to talk about their inability to cooperate from time to time with their children when it came to their demands, and about how unconditional acceptance was far from reality as well as unachievable.

The three same-sex couples that participated in this study were outstanding at giving detailed information regarding idiosyncrasies and mutual adaptation processes between them and their children, as well as providing acceptable alternatives to the inability to fulfill their children’s wishes; both characteristics were part of the fifth subtheme: *Taking the child’s perspective into account.* These aspects were also underlined by Ainsworth decades ago, as centerpieces of the quality of care organization (Ainsworth, 1967; Ainsworth et al., 1978).

In 2010, Bretherton urged that more attention should be placed on the quality of the relationship among parents because parental collaboration might depend on how mothers and fathers evaluate each other as parents (Bretherton, 2010). The sixth subtheme, *Agreeing on roles and dividing the tasks*, took into account and incorporated the idea of recognizing one another’s work and the possibility of creating agreements and supporting each other. Thus, these findings are in line with Bretherton’s affirmations.

### Limitations and Future Directions

Finding Mexican same-sex parented families with 0- to 3-year-old children willing to participate was a very hard task. From a positivist view of science, purposive and non-probabilistic sampling ought to be acknowledged as a methodological limitation in the present study. The high socioeconomic status and education of the participants by no means represents the Mexican reality; their comfortable status-quo enabled them to access assisted reproductive technologies (as the two lesbian couples did) and placement adoption (Gerardo and Noe’s case). Pathways to parenthood in Mexican LG individuals from diverse socioeconomic backgrounds, motivations for donating, and the emotions experienced as a result of donating are important areas for future research (Riggs, 2014).

Mexico City may be an icon of a progressive and cosmopolitan image of the nation, despite Mexico being a family-oriented and highly religious country, with over 83% of the population identifying with Catholicism (National Institute of Statistics Geography [INEGI], 2010). As a consequence, people with diverse sexual orientations face constant discrimination. According to Mexico City’s 2017 survey on discrimination, from a list of 41 groups subject to severe discrimination, *gays* appeared in the second place (12.1%) only after indigenous people (Consejo para Prevenir y Eliminar la Discriminación de la Ciudad de México [EDIS], 2017). The 2016 presidential proposal to introduce equal access to marriage for the whole country produced social cataclysm, including declarations from the *National Front for the Family* (Frente Nacional por la Familia), a right-wing group that opposes same-sex marriage and child adoption. This conservative wave represents a setback in the respect of human rights, since the prohibition of such rights marginalizes LG people and their children and sends a message to the wider community that same-sex families are not legally or morally acceptable (Costa and Salinas-Quiroz, 2018). To the present time, LG people are only able to openly and purposefully partake in creating legally recognized families in 12 out of 32 Mexican States, therefore, research that takes into account prejudice and discrimination against same-sex planned families must be encouraged throughout the country.

Given the aforementioned, case studies would be particularly welcomed and appreciated because they offer a means of investigating complex social situations of potential importance in understanding parenting styles and practices in unconventional contexts. This method enables a process of continuous discussion and the redefinition of research questions (Stake, 2008). Besides that, more research is needed with different methodological devices, as the interviews’ content showed a variety of very valuable elements related to the quality of care organization, such as the importance of *Play, Exploration and Experimentation*, and *Concepts Regarding Development and Learning*, among other seventeen over-arching themes which were not discussed in this study.

The diversification of both instruments and types of study, including naturalistic observations in multiple settings and occasions, as well as longitudinal designs, is now of utmost importance in order to explore the organization of quality of care in same-sex parented families.

### Final Reflections

Attachment researchers hypothesize that the quality of caregiving remains a central factor in maintaining and shaping the organization of secure-base behaviors throughout childhood. Ainsworth’s legacy lies not only in her findings and the constructs she developed, but also in the way in which she conducted developmental science (Bretherton, 2013). Inspired on her work, a collective case study design was chosen, so as to contribute to the theoretical discussion of the elements that configure the quality of care organization, and to analyze what makes some Mexican same-sex planned families unique (Clarke, 2002). Two different but complementary tools that are not commonly used in quality of care research were integrated: the Q-sort methodology and semi-structured interviews. Both the AQS and the MBQS assesses children’s and parents’ behavior, respectively, but rely solely on naturalistic observations. By adding biographic data and the reconstruction of parents’ day-to-day lives, the interviews became an opportunity to contribute to the understanding of their parenting and childrearing practices. The thematic analysis allowed researchers to identify situations, characters, temporary sequences and, overall, an argumentative narrative (Clandinin and Connelly, 2000; Bruner, 2004) about the quality of care.

Findings highlight the heuristic power of Ainsworth’s notions of quality of care, which are relevant when researching on child–caregiver attachment relationships in same-sex parented families. To conclude, it can be stated that these same-sex couples were capable of meeting the child’s best interests, providing “ordinary expectable caregiving environments” (Bowlby, 1969/1982), as they were afforded the same rights and accepted the same responsibilities as heterosexual parents. The three same-sex planned families lived in a privileged city where this is now possible. However, there is still a long way to go in order to provoke a shift in the way of thinking, so family processes can be viewed as more important than family structure regarding children’s psychological wellbeing (Golombok and Tasker, 2015).

## Author Contributions

FS-Q and FR-S: conception of the work. FS-Q, MR, PS, and VC: acquisition of data. FS-Q, PS, and VC: data analysis. FS-Q, FR-S, PC, and MR: interpretation of data. FS-Q, FR-S, and PC: drafting the manuscript. FS-Q, FR-S, PC, MR, PS, and VC: final approval of the version to be published; agreement to be accountable for all aspects of the work.

## Conflict of Interest Statement

The authors declare that the research was conducted in the absence of any commercial or financial relationships that could be construed as a potential conflict of interest.

## Footnotes

1 “*Familias Diversas*” [Diverse Families] is a civil association of LGBT parents who interact and dialog about their experiences in order to visualize their existence.

2 The exact number of members that are actually parents as well as the age of their offspring is unknown. In order to gain a membership, an invitation from a group member is needed.

## References

[B1] Ainsworth M. D. S. (1967). *Infancy in Uganda: Infant Care and the Growth of Love.* Baltimore, MD: Johns Hopkins University Press.

[B2] Ainsworth M. D. S. (1970). *Criteria for Classification of One-Year-Olds in Terms of the Balance Between Exploratory and Attachment Behavior.* Available at: http://www.psychology.sunysb.edu/attachment/measures/content/home_secure_base_patterns.pdf

[B3] Ainsworth M. D. S. Bell S. M. Stayton D. J. (1971). “Individual differences in strange situation behavior of one-year-olds,” in *The Origins of Human Social Relations*, ed. Shaffer H. R. (London: Academic Press), 17–57.

[B4] Ainsworth M. D. S. Bell S. M. Stayton D. J. (1974). “Infant-mother attachment and social development: “Socialization” as a product of reciprocal responsiveness to signals,” in *The Integration of A Child Into a Social Word*, ed. Richards P. (Cambridge: Cambridge University Press), 99–135.

[B5] Ainsworth M. D. S. Blehar M. C. Waters E. Wall S. (1978). *Patterns of Attachment.* Hillsdale, NJ: Erlbaum.

[B6] Amazonas M. C. L. A. Veríssimo H. V. Lourenço G. O. (2013). A adoção de crianças por gays [The adoption of children by gay people]. *Psicol. Soc.* 25 631–641. 10.1590/S0102-71822013000300017

[B7] Atkinson L. Niccols A. Paglia A. Coolbear J. Parker K. C. H. Poulton L. (2000). A meta-analysis of time between maternal sensitivity and attachment assessments: implications for internal working models in infancy/toddlerhood. *J. Soc. Pers. Relat.* 17 791–810. 10.1177/0265407500176005

[B8] Averett P. Nalavany B. Ryan S. (2009). An evaluation of gay/lesbian and heterosexual adoption. *Adopt. Quart.* 12 129–151. 10.1080/10926750903313278

[B9] Bakermans-Kranenburg M. J. van IJzendoorn M. H. Juffer F. (2003). Less is more: meta-analyses of sensitivity and attachment interventions in early childhood. *Psychol. Bull.* 129 195–215. 10.1037/0033-2909.129.2.195 12696839

[B10] Biblarz T. Savci E. (2010). Lesbian, gay, bisexual and transgender families. *J. Marriage Fam.* 72 480–497. 10.1111/j.1741-3737.2010.00714.x

[B11] Blake L. Carone N. Raffanello E. Slutsky J. Ehrhardt A. A. Golombok S. (2017). Gay fathers’ motivations for and feelings about surrogacy as a path to parenthood. *Hum. Reprod.* 32 860–867. 10.1093/humrep/dex026 28333218 PMC5400050

[B12] Block J. (1978). *The Q-sort Method.* Palo Alto, CA: Consulting Psychologist Press.

[B13] Bowlby J. (1969/1982). *Attachment and Loss*, Vol. 1 New York, NY: Basic Books

[B14] Braun V. Clarke V. (2006). Using thematic analysis in psychology. *Qualit. Res. Psychol.* 3 77–101. 10.1191/1478088706qp063oa

[B15] Bretherton I. (1992). The origins of attachment theory: John Bowlby and Mary Ainsworth. *Dev. Psychol.* 28 759–775. 10.1037/0012-1649.28.5.759 9489476

[B16] Bretherton I. (2010). Fathers in attachment theory and research: a review. *Early Child Dev. Care* 180 9–23. 10.1080/03004430903414661 25319747

[B17] Bretherton I. (2013). Revisiting Mary Ainsworth’s conceptualization and assessment of maternal sensitivity-insensitivity. *Attachm. Hum. Dev.* 15 460–484. 10.1080/14616734.2013.835128 24299130

[B18] Brewaeys A. Ponjaert I. Van Hall E. V. Golombok S. (1997). Donor insemination: child development and family functioning in lesbian mother families. *Hum. Reprod.* 12 1349–1359. 10.1093/humrep/12.6.1349 9222029

[B19] Bruner J. (1980). *Realidad Mental y Mundos Posibles [Mental Reality and Possible Worlds].* Barcelona: Gedisa.

[B20] Bruner J. (2004). Life as narrative. *Soc. Res.* 71 691–710.

[B21] Cadman T. Diamond P. Fearon P. (2017). Reassessing the validity of the attachment Q-sort: an updated meta-analysis. *Infant Child. Dev.* 27:e2034 10.1002/icd.2034

[B22] Careaga G. (2011). *Familias Homoparentales [Same-Gender Parents]. V Encuentro de la Disidencia Sexual.* México: Universidad Autónoma de la Ciudad de México (UACM).

[B23] Carneiro F. A. Tasker F. Salinas-Quiroz F. Leal I. Costa P. A. (2017). Are the fathers alright? A systematic and critical review of studies on gay and bisexual fatherhood. *Front. Psychol.* 8:1636. 10.3389/fpsyg.2017.01636 28983272 PMC5613122

[B24] Carone N. Baiocco R. Lingiardi V. (2017). Single fathers by choice using surrogacy: why men decide to have a child as a single parent. *Hum. Reprod.* 32 1871–1879. 10.1093/humrep/dex245 28854718

[B25] Carone N. Baiocco R. Manzi D. Antoniucci C. Caricato V. Pagliarulo E. (2018). Surrogacy families headed by gay men: relationships with surrogates and egg donors, fathers’ decisions over disclosure and children’s views on their surrogacy origins. *Hum. Reprod.* 33 248–255. 10.1093/humrep/dex362 29237004

[B26] Clandinin J. Connelly M. (2000). *Narrative Inquiry: Experience and Story in Qualitative Research.* San Francisco, CA: Jossey-Bass.

[B27] Clarke V. (2002). Sameness and difference in research on lesbian parenting. *J. Commun. Appl. Soc. Psychol.* 12 210–222. 10.1002/casp.673

[B28] Colonnesi C. Wissink I. B. Noom M. J. Asscher J. J. Hoeve M. Stams G. J. J. M. (2013). Basic trust: an attachment-oriented intervention based on mind-mindedness in adoptive families. *Res. Soc. Work Pract.* 23 179–188. 10.1177/1049731512469301

[B29] Connell R. W. (2005). *Masculinities.* Berkeley, CA: Press.

[B30] Consejo para Prevenir y Eliminar la Discriminación de la Ciudad de México [EDIS]. (2017). *Encuesta Sobre Discriminación en la CDMX [Survey About Discrimination in CDMX].* México: COPRed Available at: http://copred.cdmx.gob.mx/storage/app/uploads/public/59a/840/2d5/59a8402d50788389814688.pdf

[B31] Costa P. A. Salinas-Quiroz F. (2018). A comparative study of attitudes toward same-gender parenting and gay and lesbian rights in portugal and in Mexico. *J. Homosex.* 10.1080/00918369.2018.1519303 [Epub ahead of print]. 30265830

[B32] Costa P. A. Tasker F. (2018). “We wanted a forever family”: altruistic, individualistic, and motivated reasoning motivations for adoption among LGBTQ individuals. *J. Fam. Issues* 39 4156–4178. 10.1177/0192513X18810948

[B33] Crowl A. Ahn S. Baker J. (2008). A meta-analysis of developmental outcomes for children of same-sex and heterosexual parents. *J. GLBT Fam. Stud.* 4 385–407. 10.1080/15504280802177615

[B34] De Wolff M. S. van Ijzendoorn M. H. (1997). Sensitivity and attachment: a meta-analysis on parental antecedents of infant attachment. *Child Dev.* 68 571–591. 10.2307/1132107 9306636

[B35] Díez J. (2013). Explaining policy outcomes: the adoption of same-sex unions in Buenos Aires and Mexico City. *Compar. Polit. Stud.* 46 212–235. 10.1177/0010414012453035

[B36] Fedewa A. Black W. Ahn S. (2015). Children and adolescents with same-gender parents: a meta-analytic approach in assessing outcomes. *J. GLBT Fam. Stud.* 11 1–34. 1080/1550428X.2013.869486

[B37] Flick U. (2004). *Introducción a la Investigación Cualitativa [An Introduction to Qualitative Research].* Madrid: Morata.

[B38] Flick U. (2007). *Qualitative Research Kit: Managing Quality in Qualitative Research.* London: SAGE Publications Ltd 10.4135/9781849209441

[B39] Giraldo S. (2015). “Azares en las prácticas de paternidad de algunos hombres gay de México, D.F. y en el acercamiento a su estudio [Contingencies in parenting practices among gay men in Mexico City and in the approach to its study],” in *Familias Homoparentales en México: Mitos, Realidades y Vida Cotidiana*, ed. Medina J. A. (México, D.F.: Letra S, Sida, Cultura y Vida Cotidiana, A.C), 63–84.

[B40] Goldberg A. E. (2012). *Gay Dads: Transitions to Adoptive Fatherhood.* New York, NY: New York University Press 10.18574/nyu/9780814732236.001.0001

[B41] Golombok S. Mellish L. Jennings S. Casey P. Tasker F. Lamb M. E. (2014). Adoptive gay father families: parent-child relationships and children’s psychological adjustment. *Child Dev.* 85 456–468. 10.1111/cdev.12155 24033323 PMC4510787

[B42] Golombok S. Perry B. Burston A. Murray C. Mooney-Somers J. Stevens M. (2003). Children with lesbian parents: a community study. *Dev. Psychol.* 39 20–33. 10.1037/0012-1649.39.1.20 12518806

[B43] Golombok S. Tasker F. (2015). “Socioemotional development in changing families,” in *Handbook of Child Psychology and Developmental Science: 3-11*, ed. Lerner R. M. (Hoboken, NJ: John Wiley & Sons, Inc), 1–45. 10.1002/9781118963418.childpsy311

[B44] Grossman K. E. Bretherton I. Waters E. Grossman K. (2013). Maternal sensitivity: observational studies honoring Mary Ainsworth 100th year. *Attach. Hum. Dev.* 15 443–447. 10.1080/14616734.2013.841058 24299128

[B45] Lingiardi V. Carone N. Morelli M. Baiocco R. (2016). ‘It’s a bit too much fathering this seed’: the meaning-making of the sperm donor in Italian lesbian mother families. *Reproduct. Biomed.* 33 412–424. 10.1016/j.rbmo.2016.06.007 27377769

[B46] Medina J. A. (2015). “Familias homoparentales y adopción en México [Same gender parented families and adoption in Mexico],” in *Familias Homoparentales en México: Mitos, Realidades y vida Cotidiana*, ed. Medina J. A. (México, D.F.: Letra S, Sida, Cultura y Vida Cotidiana, A.C.), 189–206.

[B47] Mesman J. Emmen R. A. G. (2013). Mary ainsworth’s legacy: a systematic review of observational instruments measuring parental sensitivity. *Attach. Hum. Dev.* 15 485–506. 10.1080/14616734.2013.820900 24299131

[B48] Mesman J. van Ijzendoorn M. H. Bakermans-Kranenburg M. J. (2012). Unequal in opportunity, equal in process: parental sensitivity promotes positive child development in ethnic minority families. *Child Dev. Perspect.* 6 239–250. 10.1111/j.1750-8606.2011.00223.x

[B49] Mesman J. van IJzendoorn M. H. Behrens K. Carbonell O. A. Cárcamo R. A. Cohen-Paraira I. (2015). Is the ideal mother a sensitive mother? Beliefs about early childhood parenting in mothers across the globe. *Int. J. Behav. Dev.* 40 1–13. 10.1177/0165025415594030

[B50] Monteiro L. Veríssimo M. Vaughn B. E. Santos A. J. Fernandes M. (2010). The organization of children’s secure base behaviour in two-parent Portuguese families and father’s participation in child-related activities. *Eur. J. Dev. Psychol.* 7 545–560. 10.1080/17405620902823855

[B51] Moran G. Pederson D. R. Pettit P. Krupka A. (1992). Maternal sensitivity and infant-mother attachment in a developmentally delayed sample. *Infant Behav. Dev.* 15 427–442. 10.1016/0163-6383(92)80011-I

[B52] Moran G. Pederson D. R. Tarabulsy G. M. (2011). “Becoming sensitive to sensitivity: lessons learned from the development of the maternal behavior Q-Sort,” in *Maternal Sensitivity: A Critical Review for Practitioners*, eds Davis D. W. Logdson C. (Haupauge, NY: Nova Publishers).

[B53] National Institute of Statistics Geography [INEGI] (2010). *Censo de Población y Vivienda 2010 [Population and Housing Census 2010].* México: Instituto Nacional de Estadística y Geografía.

[B54] Patterson C. J. (2009). Children of lesbian and gay parents: psychology, law, and policy. *Am. Psychol.* 64 727–736. 10.1037/0003-066X.64.8.727 19899878

[B55] Patterson C. J. Tornello S. L. (2010). Gay fathers’ pathways to parenthood: international perspectives. *J. Fam. Res.* 22 103–116.

[B56] Pederson D. R. Moran G. (1995). Maternal behavior Q-set. Caregiving, cultural, and cognitive perspectives on secure-base behavior and working models: new growing points of attachment theory and research. *Monogr. Soc. Res. Child Dev.* 60 247–254. 10.2307/1166182

[B57] Pederson D. R. Moran G. (1996). Expressions of the attachment relationship outside of the strange situation. *Child Dev.* 67 915–927. 10.1111/j.1467-8624.1996.tb01773.x 8706535

[B58] Pederson D. R. Moran G. Sitko C. Campbell K. Ghesquire K. Acton H. (1990). Maternal sensitivity and the security of infant-mother attachment: a Q-sort study. *Child Dev.* 61 1974–1983. 10.2307/1130851 2083509

[B59] Perry B. Burston A. Stevens M. Golding J. Golombok S. Steele H. (2004). Children’s play narratives: what they tell us about lesbian-mother families. *Am. J. Orthopsychiatry* 74 467–479. 10.1037/0002-9432.74.4.467 15554808

[B60] Posada G. (2013). Piecing together the sensitivity construct: ethology and cross-cultural research. *Attach. Hum. Dev.* 15 637–656. 10.1080/14616734.2013.842753 24299139

[B61] Posada G. Carbonell O. A. Alzate G. Plata S. J. (2004). Through Colombian lenses: ethnographic and conventional analyses of maternal care and their association with secure base behavior. *Dev. Psychol.* 40 508–518. 10.1037/0012-1649.40.4.508 15238039

[B62] Posada G. Jacobs A. Richmond M. K. Carbonell O. A. Alzate G. Bustamante M. R. (2002). Maternal caregiving and infant security in two cultures. *Dev. Psychol.* 38 67–78. 10.1037//0012-1649.38.1.67 11806703

[B63] Posada G. Lu T. Trumbell J. Kaloustian G. Trudel M. Plata S. J. (2013). Is the secure base phenomenon evident here, there, and anywhere? A cross-cultural study of child behavior and expert’s definitions. *Child Dev.* 84 1896–1905. 10.1111/cdev.12084 23495673

[B64] Posada G. Trumbell J. Noblega M. Plata S. Peña P. Carbonell O. A. (2016). Maternal sensitivity and child secure base use in early childhood: studies in different cultural contexts. *Child Dev.* 87 297–311. 10.1111/cdev.12454 26525825

[B65] Posada G. Waters E. (2014). “El sistema de comportamiento de cuidado: sensibilidad y apoyo de base segura [The caregiving behavioral system: Sensitivity and secure base support],” in *La Teoría Del Apego: Investigación y Aplicaciones Clínicas*, eds Torres B.Gómez de Cádiz, J. M. Causadias y G. Posada (Madrid: Psimática Editorial, S.L), 75–97.

[B66] Riggs D. (2014). Lesbian mothers, gay sperm donors, and community: ensuring the well-being of children and families. *Health Sociol. Rev.* 17 226–234. 10.5172/hesr.451.17.3.226

[B67] Scholmer G. L. Belsky J. (2012). Maternal age, investment, and parent-child conflict: a mediational test of the terminal investment hypothesis. *J. Fam. Psychol.* 26 443–452. 10.1037/a0027859 22468690

[B68] Silva P. (2015). Sensibilidad Maternal y su Relacion Con la Adquisición del Lenguaje. *Estudio Exploratorio en Díadas Mama-hijo de 6 a 24 Meses en la Ciudad de Montevideo.* Unpublished master’s thesis, UdelaR, Montevideo.

[B69] Stacey J. (2006). Gay male parenthood and the decline of paternity as we knew it. *Sexualities* 9 27–55. 10.1177/1363460706060687

[B70] Stake R. (2008). “Qualitative case studies,” in *Strategies of Qualitative Inquiry*, eds Denzin N. K. Lincoln Y. S. (Thousand Oaks, CA: Sage), 119–150.

[B71] Steele M. Hodges J. Kaniuk J. Steele H. D’Agostino D. Blom I. (2007). “Intervening with maltreated children and their adoptive families: identifying attachment facilitative behaviors,” in *Attachment Theory in Clinical Work with Children: Bridging the Gap between Research and Practice*, eds Oppenheim D. Goldsmith D. F. (New York, NY: Guilford Press), 58–59.

[B72] Steffens M. C. Jonas K. J. Denger L. (2015). Male role endorsement explains negative attitudes toward lesbians and gay men among students in Mexico more than in Germany. *J. Sex Res.* 52 898–911. 10.1080/00224499.2014.966047 25369554

[B73] Stevenson-Hinde J. Shouldice A. (1995). Maternal interactions and self reports related to attachment classifications at 4.5 *years*. *Child Dev.* 66 583–596. 10.2307/1131936

[B74] Tornello S. Patterson C. (2015). Timing of parenthood and experiences of gay fathers: a life course perspective. *J. GLBT Fam. Stud.* 11 35–56. 10.1080/1550428X.2013.878681

[B75] United Nations Committee on the Rights of the Child [UNCRC] (2005). *Implementing Child Rights in Early Childhood.* Geneva: UNCRC Available at: http://tbinternet.ohchr.org/_layouts/treatybodyexternal/Download.aspx?symbolno=CRC%2fC%2fGC%2f7%2fRev.1&Lang=en

[B76] van IJzendoorn M. H. Vereijken C. M. J. L. Bakermans-Kranenburg M. J. Riksen-Walraven J. M. (2004). Assessing attachment security with the attachment Q Sort: meta-analytic evidence for the validity of the observer AQS. *Child Dev.* 75 1188–1213. 10.1111/j.1467-8624.2004.00733.x 15260872

[B77] Waters E. (1995). The attachment Q-set (version 3). Caregiving, cultural, and cognitive perspectives on secure-base behavior and working models: new growing points of attachment theory and research. *Monogr. Soc. Res. Child Dev.* 60 234–246. 10.2307/1166181

[B78] Waters E. Deane K. E. (1985). Defining and assessing individual differences in attachment relationships: Q-methodology and the organization of behavior in infancy and early childhood. *Monogr. Soc. Res. Child Dev.* 50 41–65. 10.2307/3333826

[B79] Wells G. (2011). Making room for daddies: male couples creating families through adoption. *J. GLBT Fam. Stud.* 7 155–181. 10.1080/1550428X.2011.537242

[B80] Zeller N. (1998). “La racionalidad narrativa en la investigación educativa [Narrative Rationality in Educational Research],” in *La Narrativa En la Enseñanza, el Aprendizaje y la Investigación*, ed. McEwan H. Egan K. (Buenos Aires: Amorrortu), 295–314.

